# Interpenetrated Structures for Enhancing Ion Diffusion Kinetics in Electrochemical Energy Storage Devices

**DOI:** 10.1007/s40820-024-01472-8

**Published:** 2024-07-25

**Authors:** Xinzhe Xue, Longsheng Feng, Qiu Ren, Cassidy Tran, Samuel Eisenberg, Anica Pinongcos, Logan Valdovinos, Cathleen Hsieh, Tae Wook Heo, Marcus A. Worsley, Cheng Zhu, Yat Li

**Affiliations:** 1 Department of Chemistry and Biochemistry, University of California, 1156 High Street, Santa Cruz, CA 95064 USA; 2 Lawrence Livermore National Laboratory, 7000 East Avenue, Livermore, CA 94550 USA

**Keywords:** Interpenetrated structure, 3D printing, Electrochemical energy storage, Ion diffusion length, Inter-electrode distance

## Abstract

The architectural design of electrodes offers new opportunities for next-generation electrochemical energy storage devices (EESDs) by increasing surface area, thickness, and active materials mass loading while maintaining good ion diffusion through optimized electrode tortuosity. However, conventional thick electrodes increase ion diffusion length and cause larger ion concentration gradients, limiting reaction kinetics. We demonstrate a strategy for building interpenetrated structures that shortens ion diffusion length and reduces ion concentration inhomogeneity. This free-standing device structure also avoids short-circuiting without needing a separator. The feature size and number of interpenetrated units can be adjusted during printing to balance surface area and ion diffusion. Starting with a 3D-printed interpenetrated polymer substrate, we metallize it to make it conductive. This substrate has two individually addressable electrodes, allowing selective electrodeposition of energy storage materials. Using a Zn//MnO_2_ battery as a model system, the interpenetrated device outperforms conventional separate electrode configurations, improving volumetric energy density by 221% and exhibiting a higher capacity retention rate of 49% compared to 35% at temperatures from 20 to 0 °C. Our study introduces a new EESD architecture applicable to Li-ion, Na-ion batteries, supercapacitors, etc.

**Supplementary Information:**

The online version contains supplementary material available at 10.1007/s40820-024-01472-8.

## Highlights

- A new and compact device configuration was created with two interpenetrated, individually addressable electrodes, allowing precise control over the geometric features and interactions between the electrodes.
- The interpenetrated electrode design improves ion diffusion kinetics in electrochemical energy storage devices by shortening the ion diffusion length and reducing ion concentration inhomogeneity.
- The device with interpenetrated electrodes outperformed the traditional separate electrode configuration, enhancing both volumetric energy density and capacity retention rate.

**Supplementary Information:**

The online version contains supplementary material available at 10.1007/s40820-024-01472-8.

## Graphical abstract

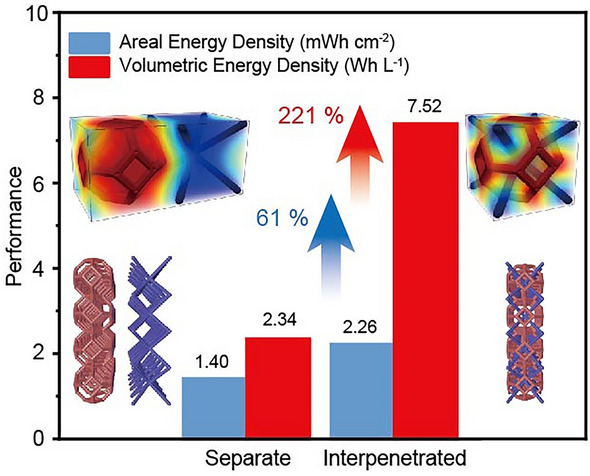

## Introduction

With the increasing global demand for electrochemical energy storage devices (EESDs), innovative approaches have been explored to improve device fabrication and performance [1–4]. Among them, 3D-printing architected electrodes represent a new direction to accommodate ultrahigh loading of active materials for increasing capacity and energy density while retaining ion diffusion efficiency [5–9]. Ion diffusion kinetics is critical for all EESDs, and it is determined by several factors [10]. First, according to the effective ionic diffusivity (*D*_eff_) equation [11], the electrode’s porosity and tortuosity are critical:

1 $${D}_{\text{eff}}=\frac{\varepsilon }{\tau }\text{D}$$

where $\varepsilon$ is the electrode porosity, $\tau$ is the electrode tortuosity, and *D* is the intrinsic diffusion coefficient. Second, the ion diffusion kinetics is also closely related to the diffusion length, as shown in the following equation:

2 $$t=\frac{{L}^{2}}{\text{D}}$$

where $t$ is the ion diffusion time, $L$ is the diffusion length, and D is the intrinsic diffusion coefficient. Architected electrodes with large porosity (surface area) and low tortuosity in three dimensions have been demonstrated in various EESDs, such as batteries or capacitors [9, 12, 13], to improve their performance [14–18]. Nevertheless, the increased thickness of 3D-printed electrodes will inevitably increase the ion diffusion length and concentration gradient between the two electrodes, resulting in sluggish ion diffusion kinetics [19, 20]. Therefore, it is highly desirable to create new electrode structures that can simultaneously enable large surface area, low tortuosity, and short inter-electrode distance, thus maintaining fast ion diffusion kinetics between electrodes at the device level.

Here, we present a new electrode configuration of interpenetrated architecture, where two independent 3D lattices with different topologies are interwoven through the same volume but still disconnected after electrode preparations. By directly designing interlocked topologies, we can precisely control the geometric features of each sublattice and, more importantly, the interactions between the two lattices. To our knowledge, this is the first time that interpenetrated lattices have been used for architectural EESDs. Significantly, ordered, periodically entangled two electrodes occupying the same free volume are close to each other throughout the whole structure regardless of its thickness. This structural characteristic is anticipated to enhance the ion diffusion kinetics during the charging and discharging processes. Specifically, we first use the digital light processing-based 3D-printing method to produce the interpenetrated lattice scaffolds composed of interlocked Kelvin cell and body-centered cell arrays followed by metallization. Then, zinc (Zn) and manganese dioxide-poly(3,4-ethylenedioxythiophene) (MnO_2_/PEDOT) are selectively electrodeposited onto each side of the sublattice, respectively, to fabricate a Zn//MnO_2_ battery as the model system [21–23]. This interpenetrated device improves the volumetric energy density by 221% compared with the conventional device in a separate electrode configuration. The interpenetrated device also achieves a better capacity retention rate (49%) than the separate configuration (35%) when the operating temperature decreases from 20 to 0 °C. Our study provides a new design perspective to improve ion diffusion kinetics in various EESDs.

## Experimental Methods

### Chemical Reagents

Commercially plant-based resin (Gray) was purchased from Elegoo to print interpenetrated lattices. Sodium hydroxide (NaOH), hydrochloric acid (HCl, 12.1 M), nickel sulfate (NiSO_4_), ammonium hydroxide (NH_3_·H_2_O), ammonium chloride (NH_4_Cl), citric acid anhydrous (C_6_H_8_O_7_), sodium dodecyl sulfate (SDS), zinc sulfate heptahydrate (ZnSO_4_·7H_2_O), boric acid (H_3_BO_3_), dimethyl sulfoxide (DMSO), and sodium sulfate (Na_2_SO_4_) were purchased from Fisher Scientific. Manganese sulfate monohydrate (MnSO_4_·H_2_O) and sodium hypophosphite (NaH_2_PO_2_·H_2_O) were purchased from Spectrum. Manganese acetate tetrahydrate (Mn(CH_3_CO_2_)_2_·4H_2_O) was purchased from Thermo Scientific. Lithium perchlorate (LiClO_4_) was purchased from ACROS. 3,4-ethylenedioxythiophene (EDOT) was purchased from TCI. Tin(II) chloride anhydrous (SnCl_2_) was purchased from Aldrich; palladium(II) chloride (PdCl_2_) was purchased from Millipore. All chemicals were used without further purification.

### 3D Printing of Interpenetrated Polymer Substrates

A commercial masked stereolithography 3D printer (Saturn 8 K, Elegoo) was used to produce parts. The Kelvin cell–body-centered cell interpenetrated structures presented in this work were generated in a commercial 3D computer graphics and computer-aided design application software (Rhinoceros 3D). All 3D-printed parts were rinsed and sonicated in isopropyl alcohol for approximately 5 min. These cleaned parts were post-cured for 1 h at 60 °C by exposing to 405-nm light (Form Cure, Formlabs). To increase the hydrophilicity of the printed polymers, parts were plasma-treated using an air plasma cleaner (Harrick Plasma) for 2 min at a radio-frequency power of 18 W.

### Preparation of Conductive Interpenetrated Structures

Metallic Ni was deposited onto the 3D-printed interpenetrated polymer substrate to form a conductive structure via electroless plating followed by electrodeposition. Before electroless plating, the polymer substrate was immersed in a 3 M NaOH aqueous solution to remove surface impurities at 45 °C for 1 h. Then, the polymer surface was metalized by an electroless plating method, which includes sensitization, activation, and plating processes. Note that part of the structural supporting pillar was covered with tapes to separate the substrate into two electrodes after metallization. First, the polymer substrate was immersed into a sensitizing solution (10 g L^−1^ SnCl_2_, 0.12 M HCl) at 45 °C for one hour, then rinsed with deionized (DI) water. Then, the sensitized substrate was immersed into an activation solution (100 mg L^−1^ PdCl_2_, 0.03 M HCl) at 45 °C for another hour and washed with DI water. Finally, the activated sample was immersed into an electroless plating solution (13 g L^−1^ NiSO_4_, 21 g L^−1^ C_6_H_8_O_7_, and 10.6 g L^−1^ NaH_2_PO_2_·H_2_O) at 70 °C for 15 min, keeping the solution at a pH of 14 by adding ammonium hydroxide (NH_3_·H_2_O). After washing with DI water, the substrate was dried under ambient conditions.

To further improve the electrical conductivity of the interpenetrated structure, a layer of Ni coating was electrodeposited onto the electroless plated substrate (working electrode) using a two-electrode system (Ni foam was used as counter and reference electrode). The deposition process was conducted in an electrolyte composed of 40 g L^−1^ NiSO_4_ and 6.4 g L^−1^ NH_4_Cl (as a surface reagent) at a constant voltage of − 2.0 V for 30 min for each electrode using a Biologic electrochemical workstation.

### Preparation of Interpenetrated Zn//MnO_2_ Devices

After Ni electroplating, the A and B electrodes in the interpenetrated structure are conductive and individually addressable. Stainless steel foils were attached to the A and B electrodes to create a contact for the following electrodeposition of active charge storage materials. Then, epoxy was used to seal the entire structural supporting part, only exposing the interpenetrated electrode region. Zn was electrodeposited on B electrode in the structure using the two-electrode system (a Zn plate was used as CE/RE) in an electrolyte composed of 127 g L^−1^ ZnSO_4_·7H_2_O, 127 g L^−1^ Na_2_SO_4_, and 20 g L^−1^ H_3_BO_3_ at a constant current of -40 mA cm^−2^ for 30 min. MnO_2_/PEDOT composite materials were electrodeposited on A electrode using a three-electrode system (a Pt mesh as CE, Ag/AgCl as RE) in an electrolyte composed of 0.01 M 3,4-ethylenedioxythiophene (EDOT), 0.1 M LiClO_4_, 0.1 M SDS, and 0.1 M Mn(CH_3_CO_2_)_2_·4H_2_O for 60 cycles. PEDOT is to provide better adhesion between MnO_2_ and Ni surfaces [24, 25]. Each deposition cycle is conducted at a constant current of 10 mA cm^−2^ for 1 min, then 0 mA cm^−2^ for 10 s. The MnO_2_/PEDOT mass loadings for all samples were ~ 20 mg cm^−2^. Finally, the three-electrode system (a Pt mesh as CE, Ag/AgCl as RE) in the electrolyte composed of 0.01 M 3,4-ethylenedioxythiophene (EDOT), 0.1 M LiClO_4_, 0.1 M SDS were applied for an additional 1 min of PEDOT electrodeposition before device electrochemical tests.

### Materials Characterization

The crystal phase of both electroless and electro-plated Ni was characterized using X-ray diffraction (XRD) (Rigaku Smartlab Powder and Thin Film Diffractometer). The morphological information and interpenetrated electrode distance were characterized using scanning electron microscopy (SEM). Surface elemental analysis was performed for the Zn anode and MnO_2_/PEDOT cathode using a Thermo Apreo SEM with Oxford Ultim Max EDS using Aztec Software.

### Electrochemical Measurements

All Zn//MnO_2_ devices’ electrochemical tests were conducted in a two-electrode configuration in a beaker cell filled with an electrolyte mixture composed of 2 M ZnSO_4_·7H_2_O, 0.1 M MnSO_4_·H_2_O in H_2_O/DMSO (v:v = 5:1) using a BioLogic electrochemical workstation. MnSO_4_ was added to improve the charge/discharge reversibility (Fig. S1). Current densities were calculated using the structures’ geometric areas. The devices’ testing voltage range is between 0.8 and 1.85 V. For Zn plating/stripping tests, the Zn//Zn symmetrical cells were performed at a fixed capacity of 1 mAh cm^−2^ and a current density of 2 mA cm^−2^.

### Simulation

To investigate the geometric differences between interpenetrated and separate devices, the diffusion equation (Eq. 3) is solved assuming a constant concentration gradient across the two electrodes.

3 $$ \partial c/\partial t = \nabla \cdot (D\nabla c) $$

where *c* is concentration, *D* is ionic diffusivity in the electrolyte. Unit cells of the interpenetrated and separate structures were, respectively, used in the simulations. Periodic boundary conditions were applied in both structures. All simulation parameters were non-dimensionalized to enhance the efficiency of numerical solutions. The system size is 100 × 100 × 100 for the interpenetrated cell and 220 × 100 × 100 for the separate cell. The dimensionless diffusivity is 10, and applied concentration difference is 1.

## Results and Discussion

**Fig. 1:**
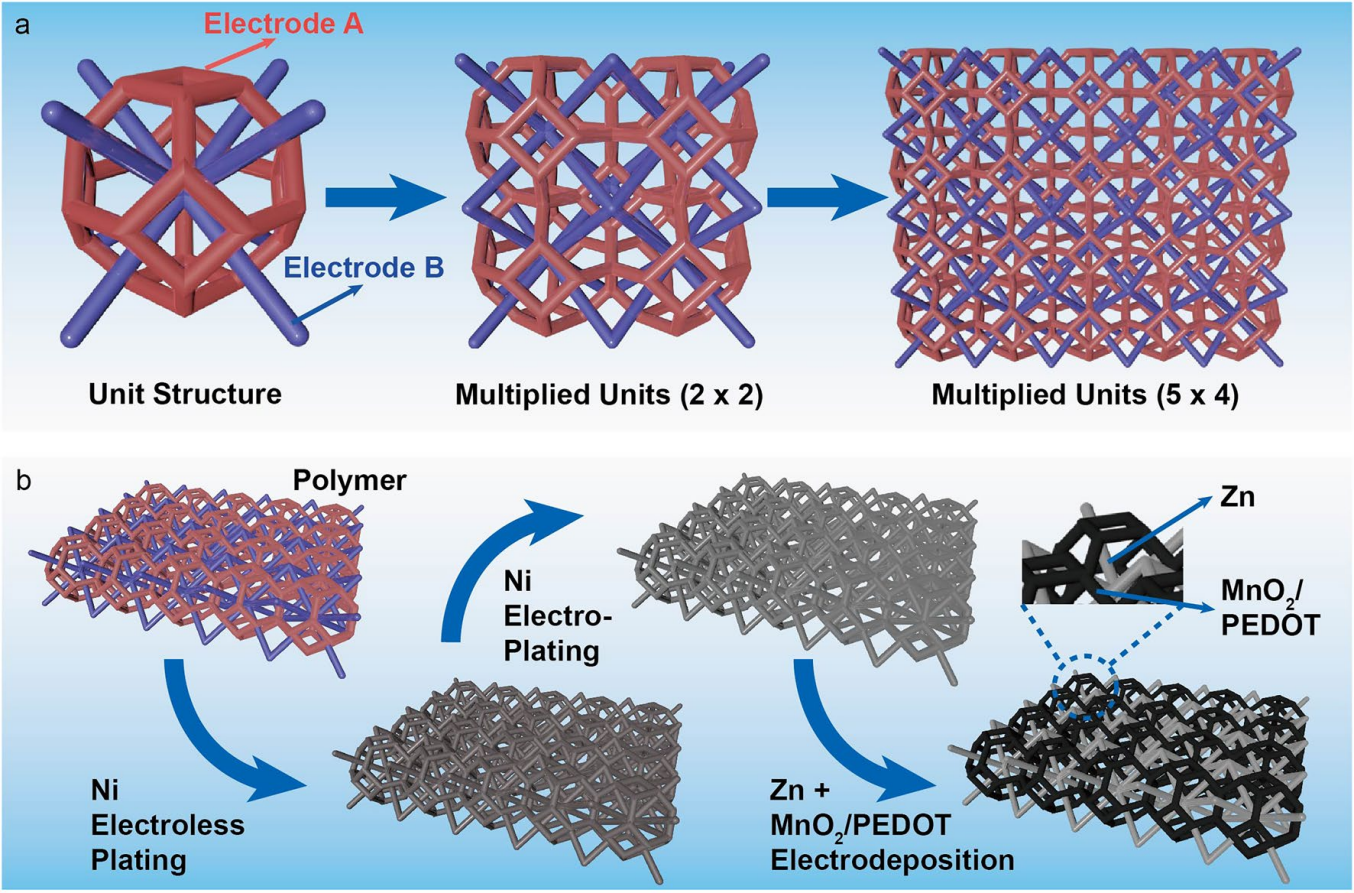
**a** Schematic illustrations of Kelvin cell–body-centered cell interpenetrated structure with single and multiple unit cells. **b** Schematic diagram showing the fabrication of an interpenetrated Zn//MnO_2_ device

The interpenetrated structure design and device fabrication are illustrated in Fig. 1. Our model system is a Kelvin cell–body-centered cell lattice structure, which contains two separate sublattice electrodes (A and B) in each unit cell (Fig. 1a). Polymeric interpenetrated structures composed of different numbers of unit cells were first printed by the stereolithography (SLA) method (Fig. 1b) using a commercially available resin as a precursor (Experimental Section). Then electroless plating was used to metallize this polymer substrate to make it conductive [26]. Specifically, the surface of polymer substrates was sensitized with Sn^2+^ ions, and then Pd nanoparticles as catalytic active sites were assembled on the polymer surface through the redox reaction between Sn^2+^ and Pd^2+^ ions via an activation process [27]:

4 $${\text{Sn}}^{2+}+{\text{Pd}}^{2+}\to {\text{Sn}}^{4+}+\text{Pd}$$

The activated substrates were further immersed into a solution mixture containing Ni^2+^ ions and a reducing agent NaH_2_PO_2_, forming a Ni–P conductive composite layer on Pd seeds [28]. An additional layer of metallic Ni was electrodeposited onto the substrate to improve the substrate's electrical conductivity further. Notably, we covered part of the electrode supporting structure during the electroless plating and electroplating, so electrodes A and B are individually addressable after the process. This conductive substrate serves as a versatile platform that enables electrodeposition of the same or different materials on the A and B electrodes. Finally, we selectively electrodeposited MnO_2_/PEDOT composite and metallic Zn on A and B electrodes, respectively. Note that adding PEDOT helps MnO_2_ deposition, and PEDOT does not contribute to the device’s capacity [29]. We use this Zn//MnO_2_ battery device as a model system to test our hypothesis on the interpenetrated EESDs.

**Fig. 2:**
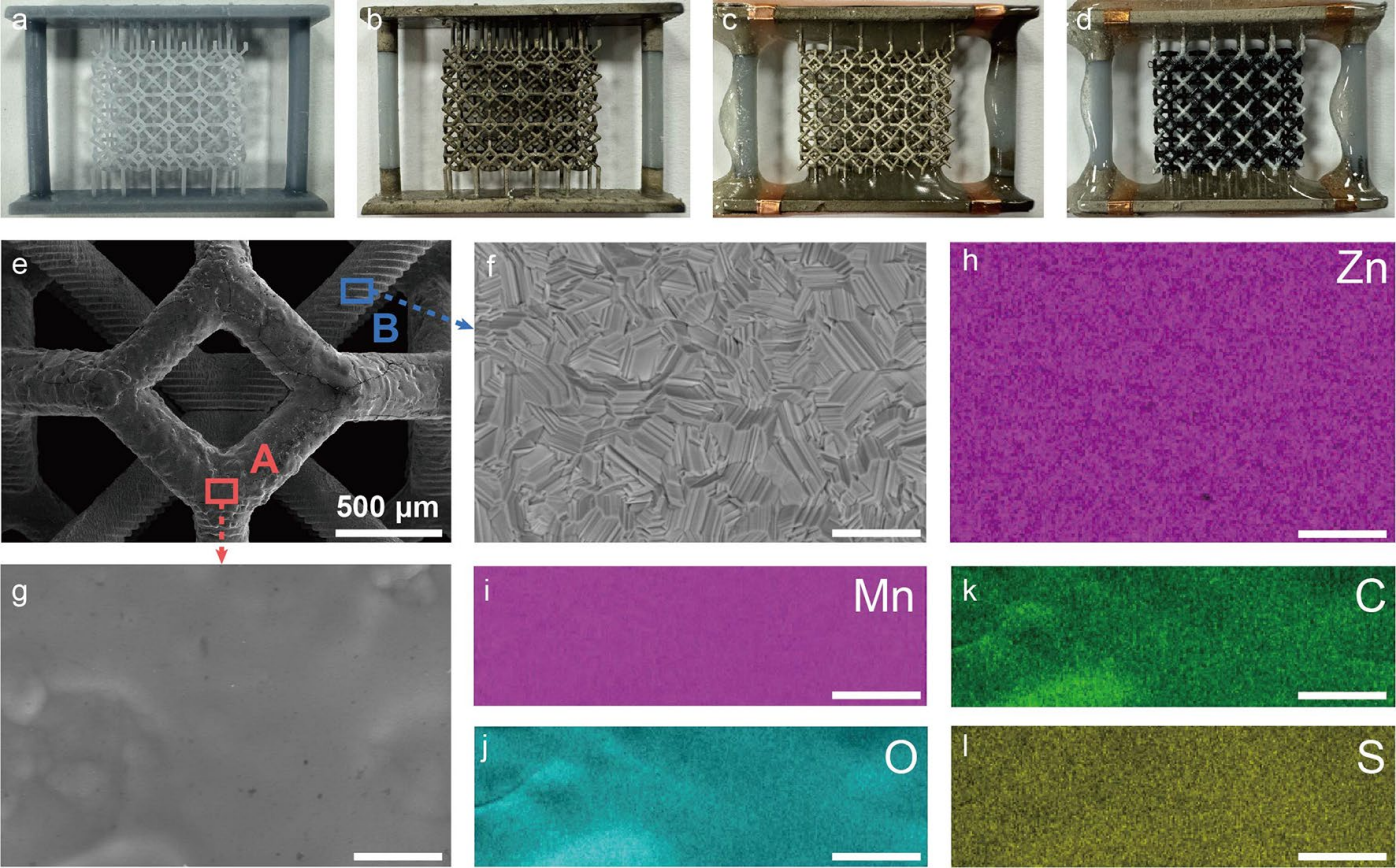
Optical images of **a** 3D-printed polymer substrate and the substrate after **b** Ni electroless plating, **c** Ni electroplating, and **d** electrodeposition of Zn and MnO_2_/PEDOT. SEM image of **e** an interpenetrated Zn//MnO_2_ structure, the magnified images of **f** Zn surface (blue box) and **g** MnO_2_/PEDOT surface (red box). EDS mapping analysis of **h** Zn surface, and MnO_2_/PEDOT surface: **i** Mn, **j** O, **k** C, and **l** S. Scale bars in **f-l** are 10 μm

Figure 2a–d shows the optical images of substrates after printing, Ni electroless plating, Ni electroplating, Zn, and MnO_2_/PEDOT electrodeposition. There is no structural deformation or shrinkage of the printed polymer substrate during these processes, which is critical for avoiding short circuit. As shown in Fig. 2d, MnO_2_/PEDOT and Zn were selectively electrodeposited on electrodes A and B without contacting each other. SEM images were collected from the substrate at different processing stages (Fig. S2). The 3D-printed polymer substrate has a smooth surface. Uniform coatings were observed after electroless and electroplating. The XRD spectrum obtained from the substrate after electroless plating suggests that the coating is metallic Ni mixed with a small amount of Ni_2_P (Fig. S3). After the Ni electroplating process, only Ni signals were observed from the substrate. We believe this electroplating improves the electrical conductivity of the substrate. The EDS elemental analysis also revealed that electrode B is fully covered with Zn coating (Fig. S4). The O to Mn atomic ratio on electrode A is approximately 2:1, as expected. The presence of C and S signals supports the success of the co-deposition of PEDOT. Energy-dispersive X-ray spectroscopy (EDS) mapping results further confirm the selective and uniform deposition of MnO_2_/PEDOT composite and Zn on electrodes A and B, respectively (Fig. 2f–m).

**Fig. 3:**
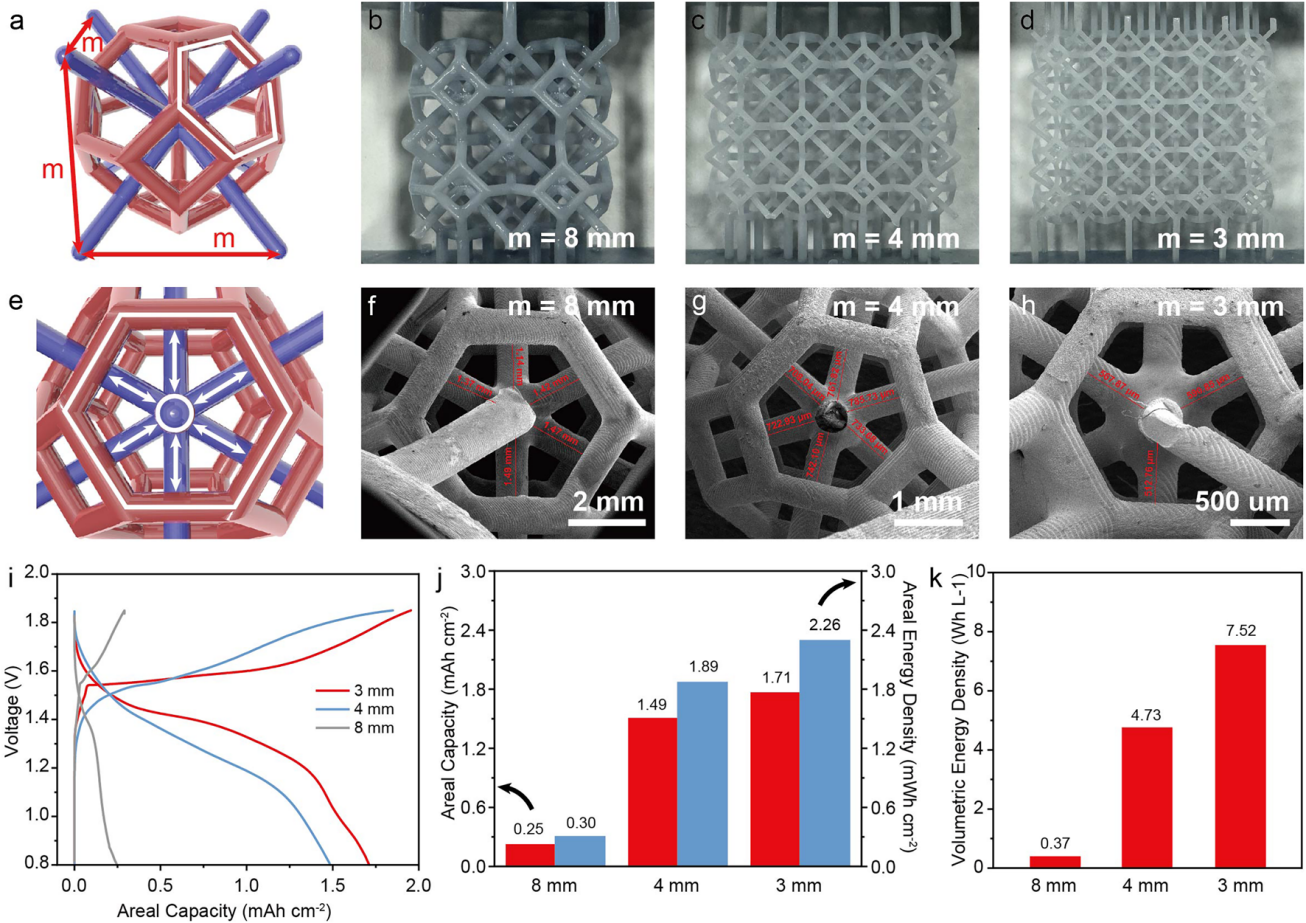
**a** Schematic illustration of a unit cell structure. Optical images of interpenetrated polymer substrates constructed with different sizes of unit cells: **b** m = 8 mm, **c** m = 4 mm, and **d** m = 3 mm. **e** A schematic diagram showing the inter-electrode distance. SEM images showing the inter-electrode distances in interpenetrated structures: **f** m = 8 mm, **g** m = 4 mm, and **h** m = 3 mm. **i** GCD curves, **j** areal capacity and energy density, and **k** volumetric energy density of interpenetrated Zn//MnO_2_ devices with different feature sizes obtained at 0.2 mA cm^−2^

We demonstrated the deposition of materials on interpenetrated structures with different feature sizes and used them to investigate the influence of size on the device’s electrochemical performances. Figure 3a illustrates the dimensions of one unit cell. Each unit fits in a cubic space with a side length of m. Figure 3b–d shows the optical images of printed interpenetrated structures with various m values (m = 8, 4, 3 mm). Reducing feature size increases the number of unit cells per unit area (Table S1, Supporting Information) and shortens the inter-distance between electrodes A and B (Fig. 3e). SEM images (Fig. 3f–h) obtained from a Ni electro-plated substrate show that the average inter-electrode distance decreases from ~ 1380 to ~ 557 μm when the m value reduces from 8 to 3 mm. There is no shortage in any of these samples. Figure 3i shows the electrochemical galvanostatic charge–discharge (GCD) curves of the interpenetrated Zn//MnO_2_ battery devices with different feature sizes collected at 0.2 mA cm^−2^. All samples have similar mass loading of MnO_2_/PEDOT (~ 20 mg cm^−2^). The device’s capacity increases as the feature size decreases. Among them, the 3 mm device exhibits the highest areal capacity of 1.71 mAh cm^−2^ and areal energy density of 2.26 mWh cm^−2^ (Fig. 3j). The smaller feature size also allows a more compact design, whereas the 3 mm device’s volume is only ~ 26.4% of the 8 mm device (Table S1). As a result, the 3 mm device achieves a much higher volumetric energy density of 7.52 Wh L^−1^ (Fig. 3k). Notably, the 3 mm device also has the lowest solution resistance (*R*_s_) and charge transfer resistance (*R*_ct_) (Fig. S5). These findings suggest that the enhanced capacity in the 3 mm device is not only related to the increased surface area but also the improved ion diffusion kinetics due to shortened inter-electrode distance.

**Fig. 4:**
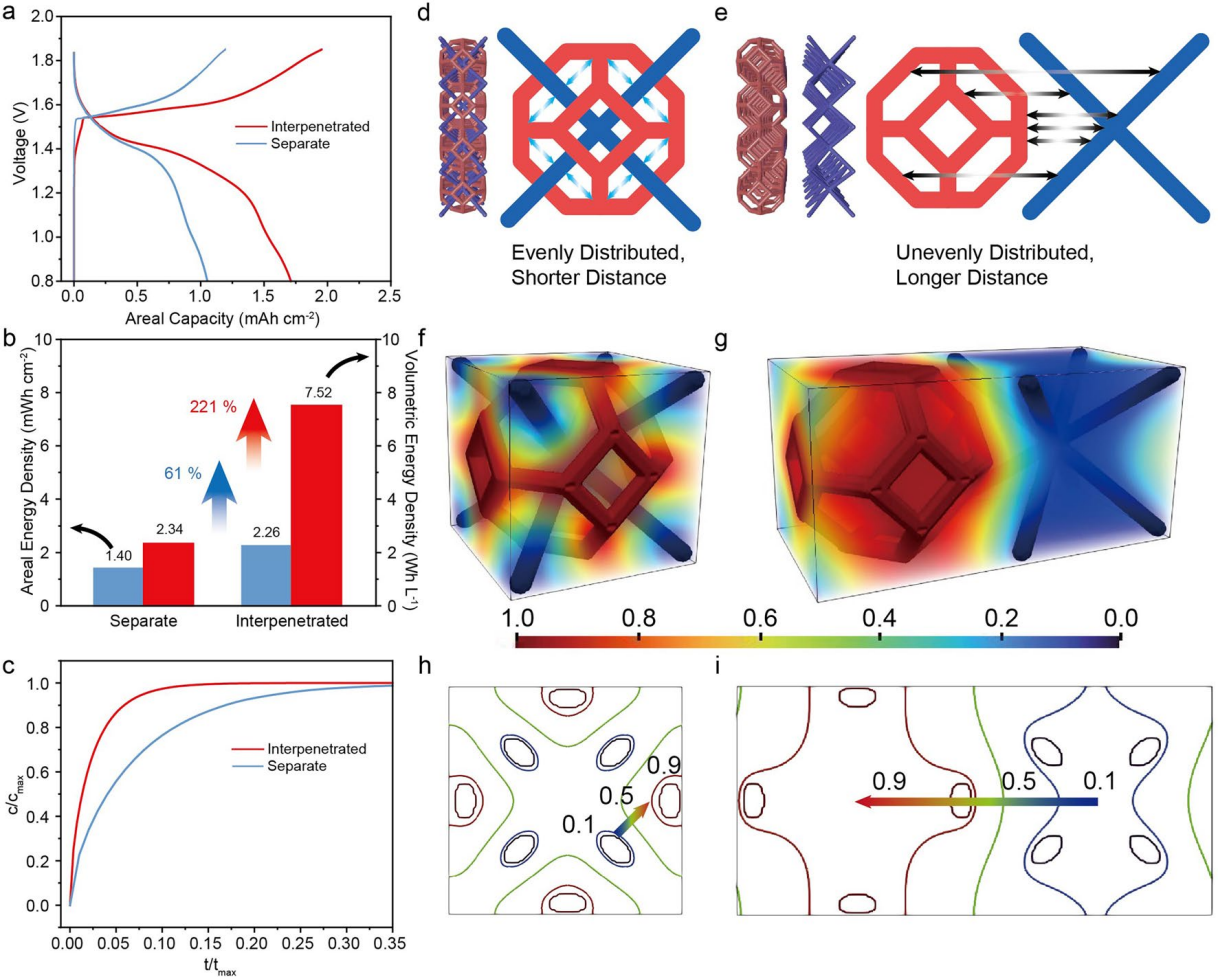
**a** GCD curves, and **b** areal and volumetric energy density of interpenetrated and separate devices under 0.2 mA cm^−2^. **c** Simulated ion diffusion time in interpenetrated and separate devices. Illustration of ion diffusion pathway length in **d** interpenetrated and **e** separate structures. Normalized equilibrium concentrations in **f** interpenetrated and **g** separate structures. **h** and **i** are cross sections of **f** and **g**, the depth intercept is 50%

To better understand the effect of shortening inter-electrode distance on device performance, we fabricate a control 3 mm device with a conventional sandwich configuration of separate electrodes (Fig. S6). The separation between the Zn anode and MnO_2_ in this control sample is ~ 5 mm. The two devices have the same geometric area (1.8 cm^2^), while the interpenetrated device’s volume (0.54 cm^3^) is half the control. CV scans (Fig. S7) show a smaller redox potential polarization in the interpenetrated device, which indicates the improved ion diffusion kinetics. In addition, GCD analysis shows that the interpenetrated device exhibits a lower overpotential for both charging and discharging processes than the control device (Fig. S8). The interpenetrated Zn//MnO_2_ device operates stably for 9 cycles at a low rate of 0.2 mA cm^−2^ (~ 0.1 C, over a week), with stable resistance after low-rate cycling (Fig. S9). Additionally, the EIS spectrum of this device shows lower *R*_s_, *R*_ct_, and both charge transfer-controlled and mass transfer-controlled *R*_d_ (Fig. S10) compared to the control sample. These improvements indicate the shorter electrode distance enhances ion diffusion kinetics. Therefore, the interpenetrated device has a substantially higher areal capacity than the control device (Fig. 4a), improving the areal energy density by 61% (Fig. 4b). Due to the compact design, the interpenetrated device boosts the volumetric energy density by 221%. The smaller electrochemical polarization and the substantially improved energy density reflect faster ion diffusion kinetics in the interpenetrated device as a result of shortening the inter-electrode distance.

In addition to the reduced ion diffusion length, the interpenetrated design decreases the inhomogeneity of ion concentration in the device structure because of the more uniform inter-electrode distance. To elucidate the impact of geometry on ionic transport, we analyzed a unit cell from both the interpenetrated and separate electrode configurations. By numerically solving the diffusion equation within the electrolyte under a consistent concentration gradient across the electrodes, we simulated the average ion concentration evolution in the electrolyte until the equilibrium state was reached. As shown in Fig. 4c, the interpenetrated device exhibits significantly faster ion diffusion kinetics, demonstrated by approximately one-third of the relaxation time of its separated counterpart to reach equilibrium. This is because the interpenetrated structure offers a shorter and more homogeneous ion diffusion distance (Fig. 4d, e). Figure 4f, g illustrates the normalized equilibrium ion concentration distribution across the cell, while Fig. 4h, i presents the iso-concentration contours at one specific cross section. Notably, the interpenetrated device ensures a steep transition from high to low concentration across every electrode ligament (see the arrow in Fig. 4h). In contrast, the separate device exhibits a smooth transition from high to low concentration across the entire electrode to another, treating the entire electrode structure as a singular concentration source (see the arrow in Fig. 4i), suggesting inefficient geometry exploitation. This becomes more evident by analyzing the entire profiles of the iso-concentration contours in Fig. 4h, i. The contours in the interpenetrated device closely encircle the electrode ligaments, implying superior spatial utilization and more effective ion transport between electrodes. Conversely, the contours in the separate device encompass the entire electrode structure, indicating that the interpenetrated structure optimizes 3D space usage and enhances ion transport efficiency.

**Fig. 5:**
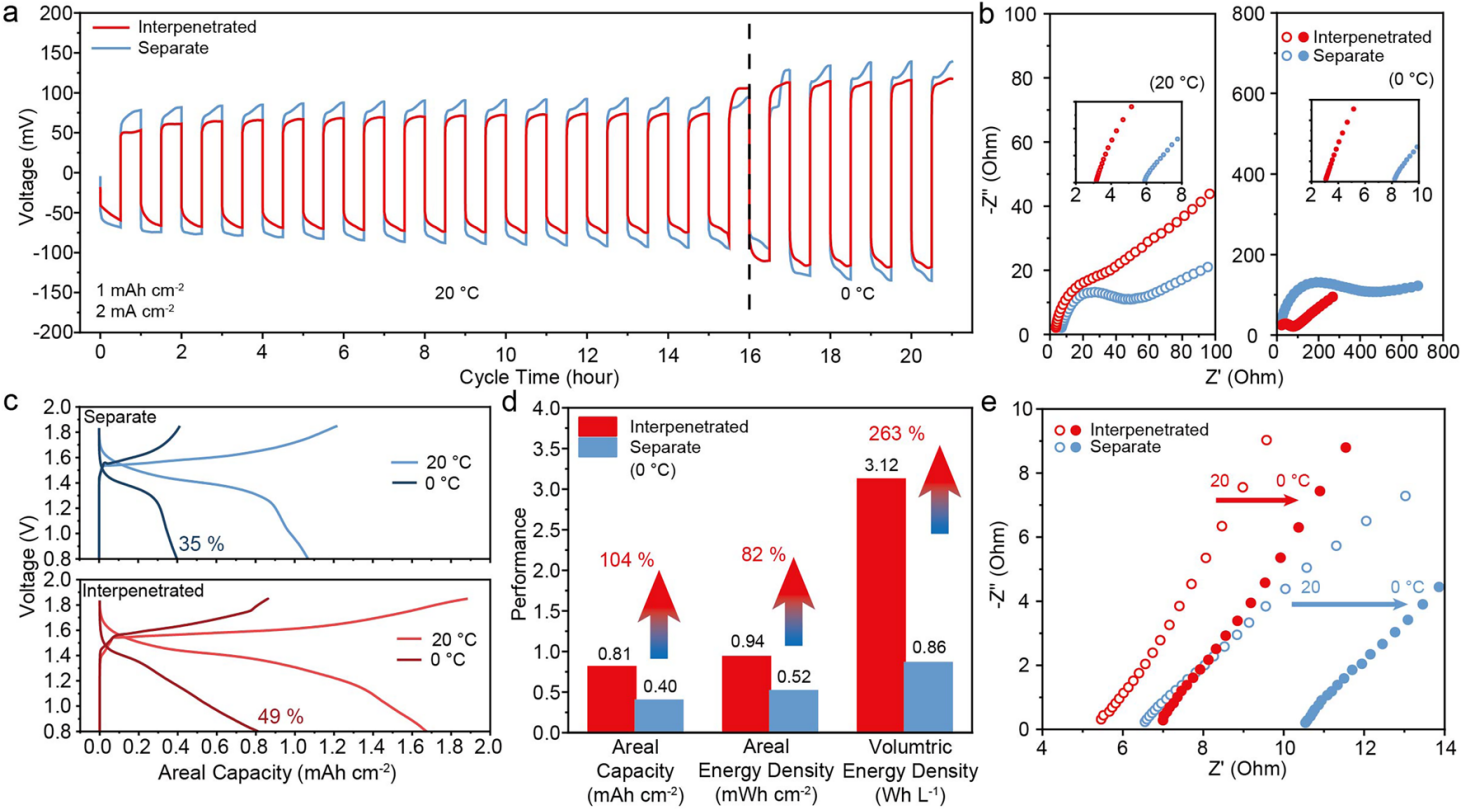
**a** Zn stripping/plating behavior observed at 2 mA cm^−2^ for 1 mAh cm^−2^ under 20 and 0 °C. **b** EIS data of the Zn//Zn symmetric cells. Inset figures are the magnified spectra in the high-frequency region. **c** GCD curves of Zn//MnO_2_ devices in interpenetrated and separate configurations obtained at 20 and 0 °C. **d** A histogram compares the areal capacity, areal energy density, and volumetric energy density of the two device configurations. **e** EIS of the Zn//MnO_2_ devices of the two configurations obtained at 20 and 0 °C

The interpenetrated structural design can be even more useful for low-temperature applications, where sluggish ion diffusion is a major challenge. Therefore, we also tested the performance of interpenetrated and separate devices at 0 °C. The electrolyte remains in the liquid phase at this temperature (Fig. S11). We started the testing with Zn//Zn symmetric cells. Figure 5a compares the Zn metal stripping/plating behavior of the two device configurations under 20 and 0 °C. It is clear that the interpenetrated structure has consistently lower polarization potentials and a more stable and smooth stripping/plating profile (Fig. S12) than that of the separate electrode design under both temperatures. Furthermore, the Zn//Zn symmetric cells in the two device configurations show considerably different electrochemical impedance spectroscopy (EIS) behaviors (Fig. 5b). Although their charge transfer resistances (*R*_ct_) are similar at 20 °C, the interpenetrated structure exhibits lower solution resistance (*R*_s_) and mass transfer resistance (*R*_d_). At 0 °C, the separate structure shows a notably increased *R*_ct_ value (~ 400 Ohm) compared with the interpenetrated design (~ 80 Ohm). Moreover, the interpenetrated structure’s *R*_s_ values remain almost unchanged (~ 3 Ohm), while the separate configuration shows a slightly increased *R*_s_ at 0 °C, from ~ 6 to ~ 8 Ohm. The interpenetrated device’s improved low-temperature performance can be attributed to the more efficient ion diffusion and uniform ion concentration distribution by shortening the inter-electrode distances.

We also extended the low-temperature testing to Zn//MnO_2_ battery devices. Figure 5c shows that the interpenetrated device can retain 49% of its areal capacity when the temperature decreases from 20 to 0 °C. The retention rate is higher than that of the separate device (35%). Benefit from the improved ion diffusion kinetics and the more compact design, the interpenetrated device shows impressive enhancement over the separate device at 0 °C; specifically, it improves areal capacity by 104%, areal energy density by 82%, and volumetric energy density by 263% (Fig. 5d). Furthermore, the *R*_s_ of the interpenetrated device only slightly increases from 5.4 to 6.9 Ohm, and these values are consistently lower than that of the separate device configuration (Fig. 5e). These results again support the significance of the interpenetrated structures in improving ion diffusion kinetics.

## Conclusions

In summary, we demonstrated a general strategy for fabricating novel interpenetrated structures for EESDs. The approach using SLA-based 3D printing of polymer substrates followed by metallization and electrodeposition of energy materials offers great flexibility in structural design/complexity and materials options. For instance, in our model system (Zn//MnO_2_ battery), we demonstrated the successful deposition of metals (Ni and Zn), semiconductor (MnO_2_), and polymer (PEDOT) on the interpenetrated structure. The entire device fabrication process does not require thermal treatment, which prevents structural deformation. Significantly, the interpenetrated device brings two electrodes in close proximity in all dimensions without causing short circuit in the absence of a separator. As a result, the interpenetrated device has a more compact design with substantially shortened and evenly distributed inter-electrode distances compared to the conventional design with two separate electrodes. Our results on Zn//Zn symmetric cells and Zn//MnO_2_ battery devices concluded that the interpenetrated (vs. separate) design greatly improves the ion diffusion kinetics, especially at a lower temperature (0 °C), and increases the volumetric energy density by 263%. Our findings offer new insights in designing EESDs.

## Supplementary Information

Below is the link to the electronic supplementary material.

Supplementary file1 (DOCX 13644 KB)
