# Supplementary material for: Interpenetrated Structures for Enhancing Ion Diffusion Kinetics in Electrochemical Energy Storage Devices
Source: Nanomicro Lett. 2024 Jul 25;16:255. doi: 10.1007/s40820-024-01472-8 (PMC11272760; doi:10.1007/s40820-024-01472-8)
Supplement: Supplementary file 1 — Supplementary file1 (DOCX 13644 KB) [file 40820_2024_1472_MOESM1_ESM.docx]

Supporting Information for

**Interpenetrated Structures for Enhancing Ion Diffusion Kinetics in Electrochemical Energy Storage Devices**

Xinzhe Xue^1^, Longsheng Feng^2^, Qiu Ren^1^, Cassidy Tran^1^, Samuel Eisenberg^1^, Anica Pinongcos^1^, Logan Valdovinos^1^, Cathleen Hsieh^1^, Tae Wook Heo^2^, Marcus A. Worsley^2,^ *, Cheng Zhu^2,^ *, Yat Li^1,^ *

^1^Department of Chemistry and Biochemistry, University of California, 1156 High Street, Santa Cruz, California 95064, United States of America

^2^Lawrence Livermore National Laboratory, 7000 East Avenue, Livermore, California 94550, United States of America

*Corresponding authors. E-mail: [worsley1@llnl.gov](mailto:worsley1@llnl.gov) (Marcus A. Worsley); [zhu6@llnl.gov](mailto:zhu6@llnl.gov) (Cheng Zhu); [yatli@ucsc.edu](mailto:yatli@ucsc.edu) (Yat Li)

**Supplementary Figures**


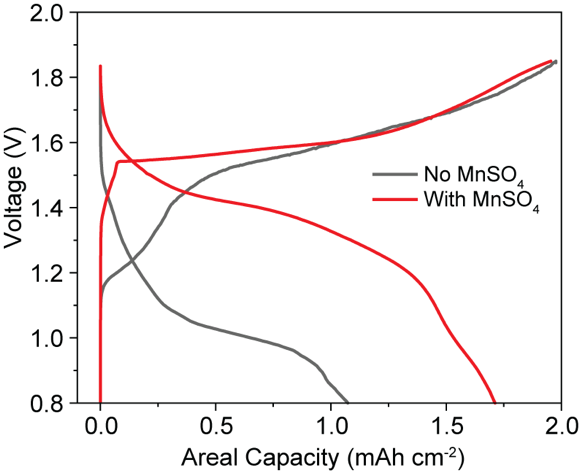


**Fig. S1** GCD curves of 3 mm interpenetrated Zn//MnO_2_ devices obtained at 0.2 mA cm^-2^ in electrolytes with and without MnSO_4_


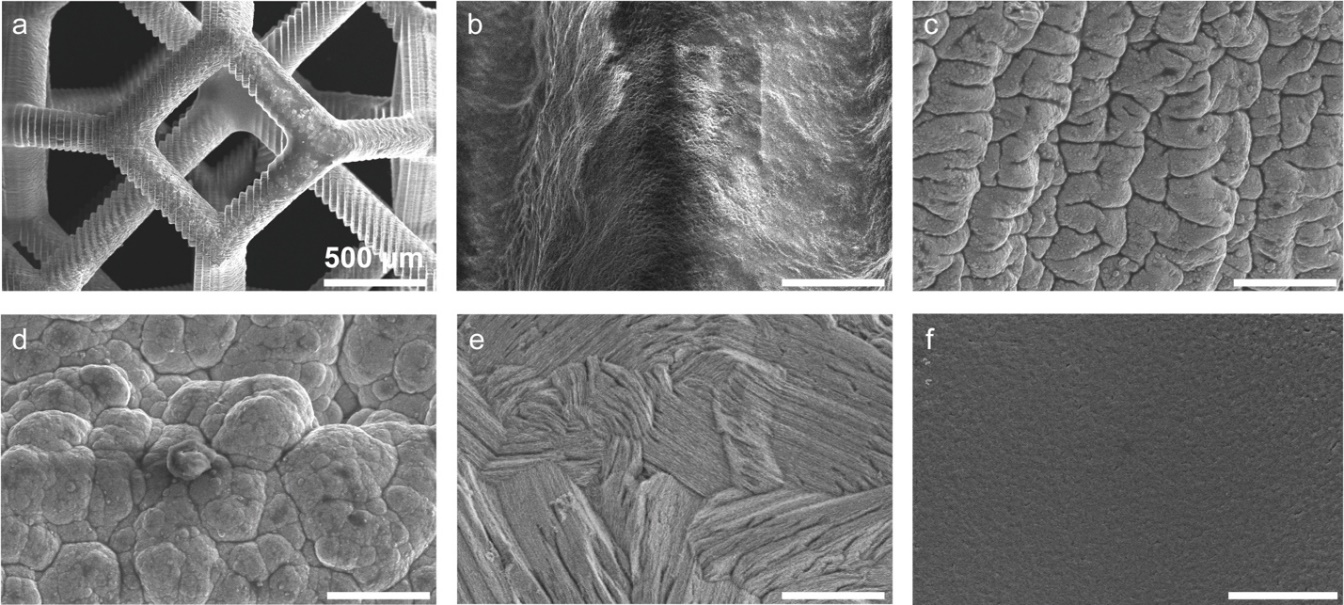


**Fig. S2** SEM images of **a** a 3D-printed interpenetrated polymer substrate, and magnified images of **b** the bare substrate and after **c** Ni electroless plating, **d** Ni electro-plating, **e** Zn electro-plating, **f** electrodeposition of MnO_2_/PEDOT surface. Scale bars in **b-f** are 10 μm


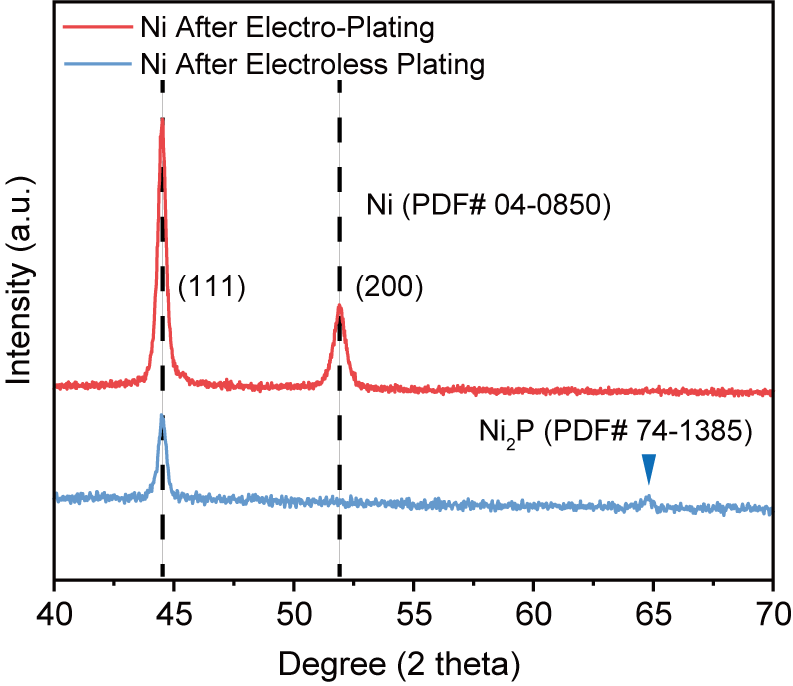


**Fig. S3** XRD patterns of an interpenetrated polymer substrate after Ni electroless (blue) and electro-plating (red). Dashed lines highlight the peak position of Ni (111) and (200). The arrow highlights the peak position of Ni_2_P


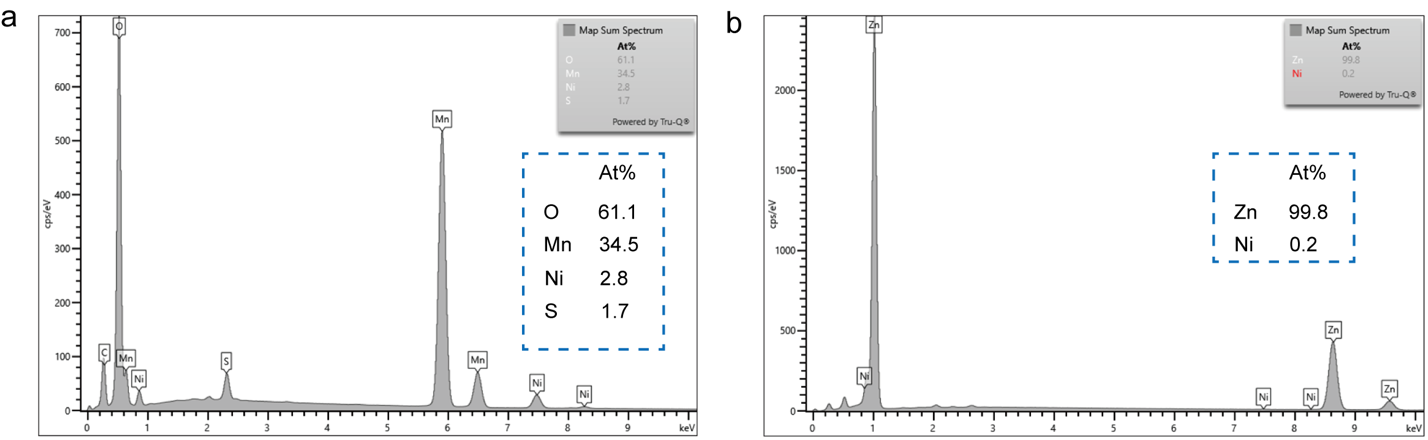


**Fig. S4** EDS elemental analysis of **a** MnO_2_/PEDOT cathode and **b** Zn anode

**Table S1** Structural data of interpenetrated devices with different feature sizes

| **m value / mm** | **Number of unit cells** | **Geometric Area / cm^2^** | **Unit Density / cm^-2^** | **Average Inter-electrode Distance / μm** | **Volume / cm^3^** | **Normalized Volume Ratio / %** |
| --- | --- | --- | --- | --- | --- | --- |
| 8 | 4 | 2.56 | 1.56 | 1380 | 2.048 | 100 |
| 4 | 12 | 1.92 | 6.25 | 742.38 | 0.768 | 37.5 |
| 3 | 15 | 1.8 | 8.33 | 557.16 | 0.54 | 26.4 |


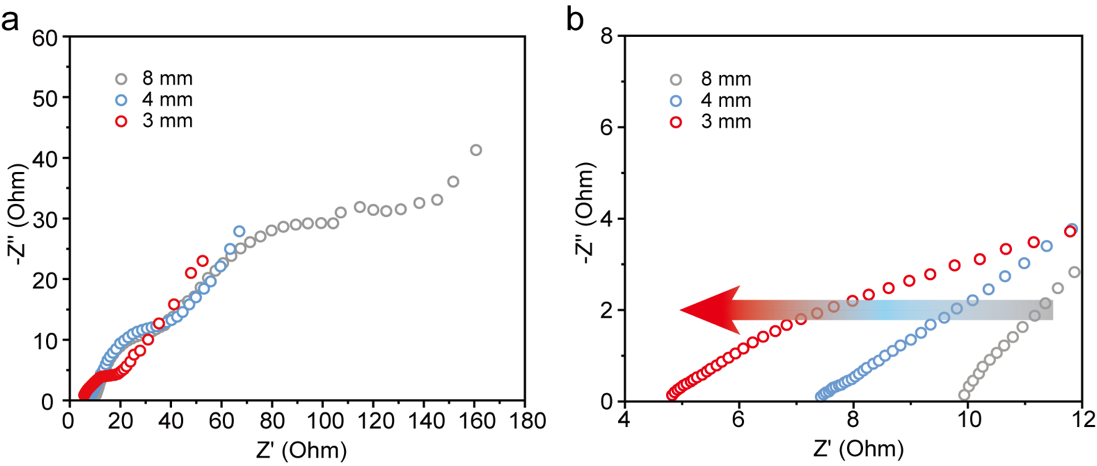


**Fig. S5** **a** Electrochemical impedance spectra of Zn//MnO_2_ devices with different feature sizes, and **b** Magnified view of the spectra in the high-frequency region


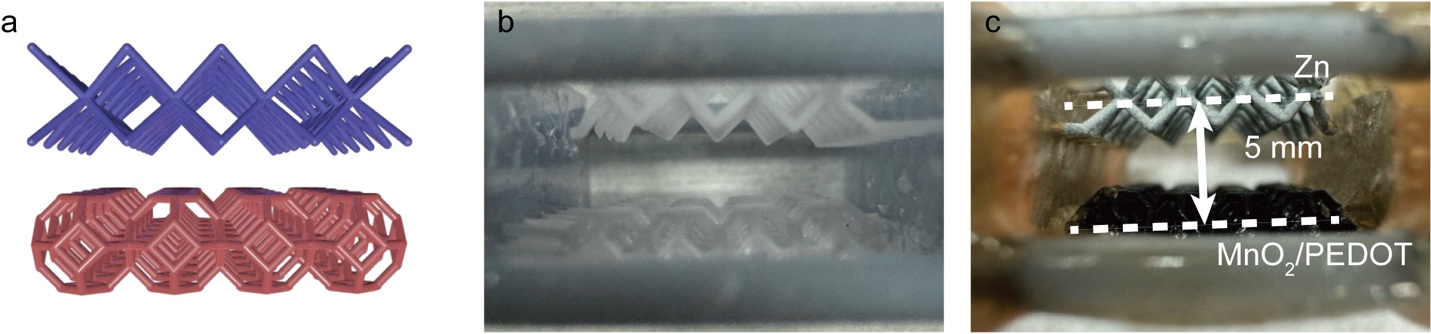


**Fig. S6 a** Schematic illustration and optical images of the separate electrodes **b** before and **c** after the deposition of Zn and MnO_2_/PEDOT


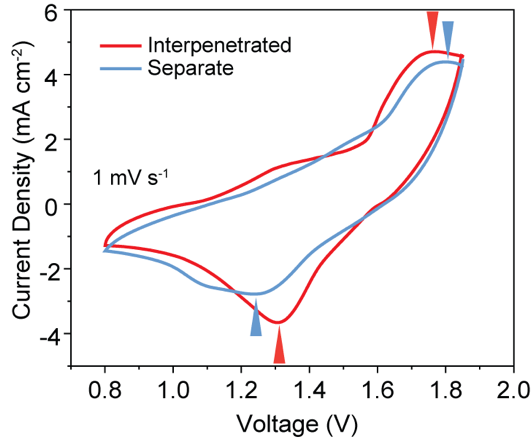


**Fig. S7** CV scans of both separate and interpenetrated devices collected at 1 mV s^-1^


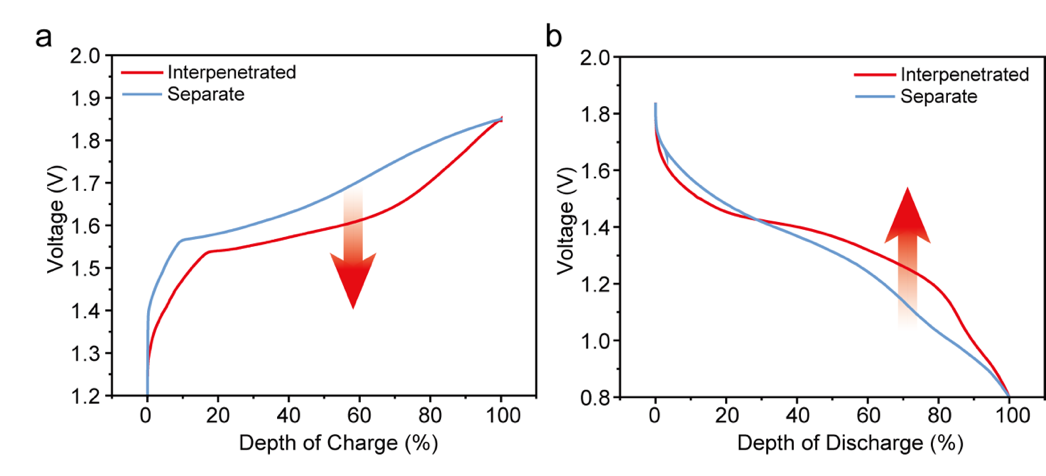


**Fig. S8** Normalized **a** charging and **b** discharging profiles of interpenetrated and separate device configurations via charging and discharging capacity collected at 0.2 mA cm^-2^


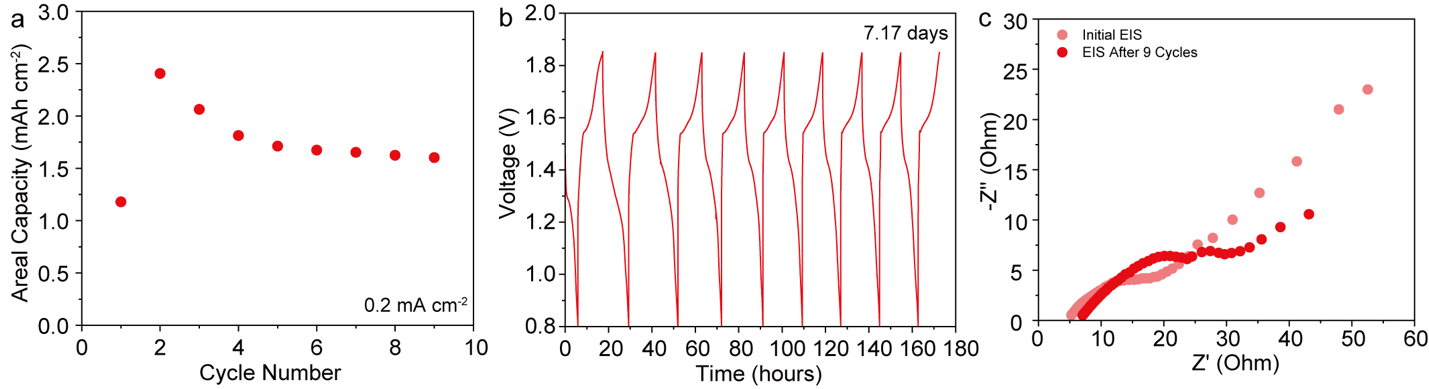


**Fig. S9 a** Cycling stability and **b** time of a 3 mm device obtained at 0.2 mA cm^-2^. **c** EIS spectra collected from the device before and after cycling test


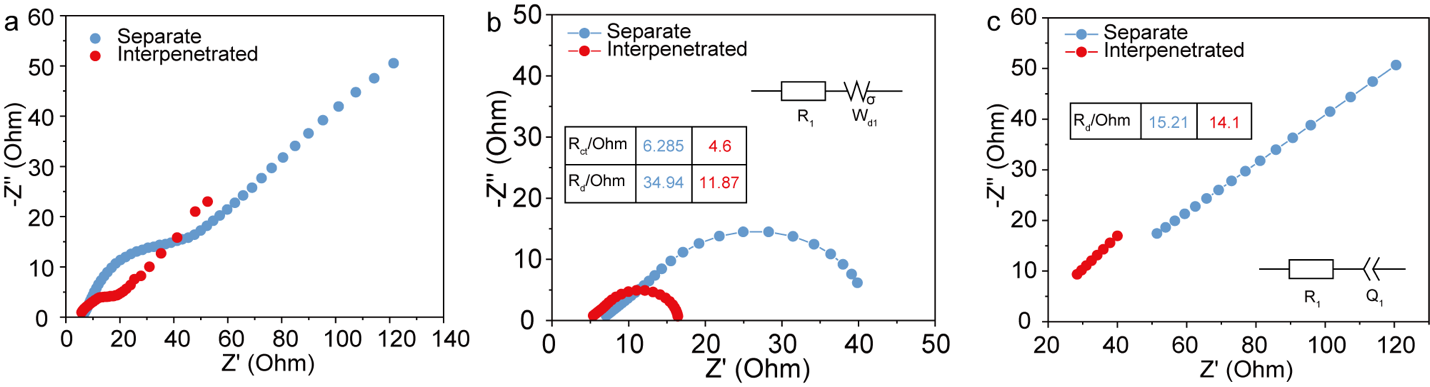


**Fig. S10** **a** EIS spectra of Zn//MnO_2_ devices with separate and interpenetrated configurations. Fitted data for **b** charge transfer controlled high frequency region, and **c** mass transfer controlled low frequency zones. The inset Figures are fitting equivalent circuits for lower and higher frequencies. The inset tables are **b** solid-state charge transfer controlled R_ct_, R_d_, and **c** mass transfer-controlled ion diffusion resistance R_d_


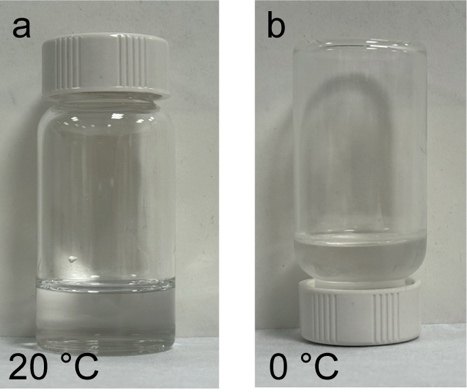


**Fig. S11** Digital images showing the electrolyte under **a** 20 °C and **b** 0 °C. The electrolyte is composed of 2 M ZnSO_4_, 0.1 M MnSO_4_ in H_2_O/DMSO (v : v = 5 : 1)


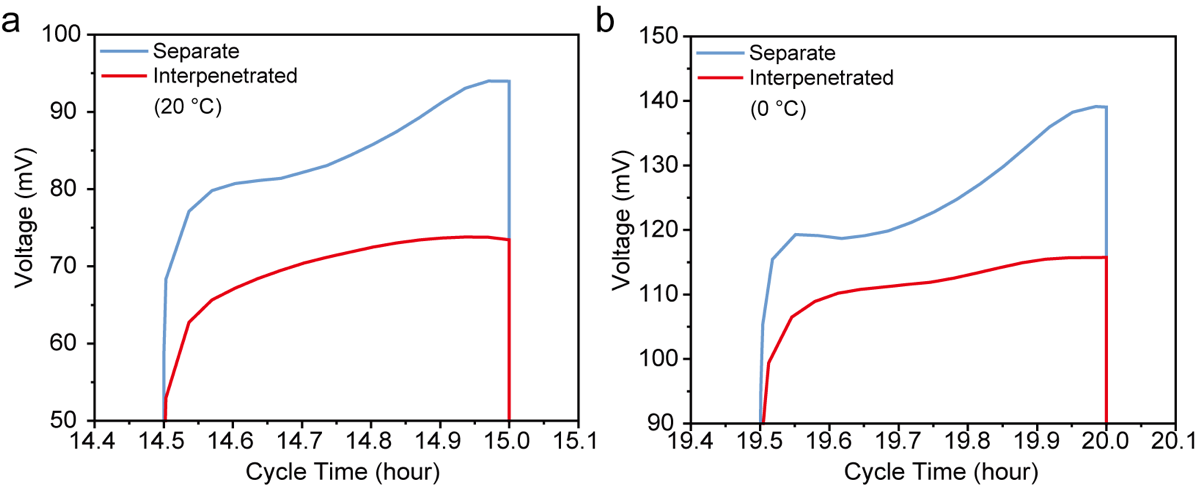


**Fig. S12** Representative Zn stripping/plating overpotentials of the interpenetrated and separate devices under **a** 20 °C and **b** 0 °C are selected for comparison

**Table S2** Performance comparisons between 2D and 3D Zn//MnO_2_ battery devices

| **Device Configurations** | **Electrolyte** | **Capacity / mAh cm^-2^** | **Device Area / cm^2^** | **Device thickness / cm** | **Device Volume / cm^3^** | **References** |
| --- | --- | --- | --- | --- | --- | --- |
| Zn//3D MnO_2_@nickel nanocone array | 2 M ZnSO_4_ + 0.2 M MnSO_4_ aq. | 3.959 | 0.48 | 7.4$\times$10^-3^ | 3.55$\times$10^-3^ | [S1] |
| Interdigitated 3D Zn//MnO_2_ battery | 2 M ZnSO_4_ + 0.1 M MnSO_4_ in PVA gel | 0.215 | 1 | 5$\times$10^-3^ | 5$\times$10^-3^ | [S2] |
| Quasi-solid-state 2D Zn//MnO_2_ battery | 3 M LiCl + 2 M ZnCl_2_ + 0.4 M MnSO_4_ in PVA gel | 2.2 | 0.5 | 0.08 | 0.04 | [S3] |
| 2D Zn//δ-MnO_2_ | 2 M ZnSO_4_ + 0.1 M MnSO_4_ in PAM gel | 0.58 | - | - | - | [S4] |
| 2D Zn//MnO_2_@CNT | 3 M LiCl + 2 M ZnCl_2_ + 0.4 M MnSO_4_ in PVA gel | 1.4 | 1 | 0.08 | 0.08 | [S5] |
| **Interpenetrated Zn//MnO_2_ battery** | **2 M ZnSO_4_ + 0.1 M MnSO_4_ aq.** | **1.71** | **1.8** | **0.3** | **0.54** | **This work** |

**Supplementary References**

1. W. Lai, Y. Wang, Z. Lei, R. Wang, Z. Lin, C.-P. Wong, F. Kang, C. Yang. High performance, environmentally benign and integratable Zn//MnO_2_ microbatteries. Journal of Materials Chemistry A **6**(9), 3933-3940 (2018). [https://doi.org/10.1039/C7TA10936A](about:blank)
2. H. Wang, R. Guo, H. Li, J. Wang, C. Du, X. Wang, Z. Zheng. 2d metal patterns transformed from 3d printed stamps for flexible Zn//MnO_2_ in-plane micro-batteries. Chemical Engineering Journal **429**(132196 (2022). [https://doi.org/https://doi.org/10.1016/j.cej.2021.132196](about:blank)
3. Y. Zeng, X. Zhang, Y. Meng, M. Yu, J. Yi, Y. Wu, X. Lu, Y. Tong. Achieving ultrahigh energy density and long durability in a flexible rechargeable quasi-solid-state Zn–MnO_2_ battery. Advanced Materials **29**(26), 1700274 (2017). [https://doi.org/https://doi.org/10.1002/adma.201700274](about:blank)
4. D. Wang, L. Wang, G. Liang, H. Li, Z. Liu, Z. Tang, J. Liang, C. Zhi. A superior δ-MnO_2_ cathode and a self-healing Zn-δ-MnO_2_ battery. ACS Nano **13**(9), 10643-10652 (2019). [https://doi.org/10.1021/acsnano.9b04916](about:blank)
5. X. Zhang, S. Wu, S. Deng, W. Wu, Y. Zeng, X. Xia, G. Pan, Y. Tong, X. Lu. 3d cnts networks enable MnO_2_ cathodes with high capacity and superior rate capability for flexible rechargeable Zn–MnO_2_ batteries. Small Methods **3**(12), 1900525 (2019). [https://doi.org/https://doi.org/10.1002/smtd.201900525](about:blank)
